# Double-Bottom Chaotic Map Particle Swarm Optimization Based on Chi-Square Test to Determine Gene-Gene Interactions

**DOI:** 10.1155/2014/172049

**Published:** 2014-05-07

**Authors:** Cheng-Hong Yang, Yu-Da Lin, Li-Yeh Chuang, Hsueh-Wei Chang

**Affiliations:** ^1^Department of Electronic Engineering, National Kaohsiung University of Applied Sciences, Kaohsiung 80778, Taiwan; ^2^Department of Chemical Engineering and Institute of Biotechnology and Chemical Engineering, I-Shou University, Kaohsiung 84001, Taiwan; ^3^Institute of Medical Science and Technology, National Sun Yat-sen University, Kaohsiung, 80708, Taiwan; ^4^Department of Biomedical Science and Environmental Biology, Translational Research Center, Cancer Center, Kaohsiung Medical University Hospital, Kaohsiung Medical University, Kaohsiung, Taiwan

## Abstract

Gene-gene interaction studies focus on the investigation of the association between the single nucleotide polymorphisms (SNPs) of genes for disease susceptibility. Statistical methods are widely used to search for a good model of gene-gene interaction for disease analysis, and the previously determined models have successfully explained the effects between SNPs and diseases. However, the huge numbers of potential combinations of SNP genotypes limit the use of statistical methods for analysing high-order interaction, and finding an available high-order model of gene-gene interaction remains a challenge. In this study, an improved particle swarm optimization with double-bottom chaotic maps (DBM-PSO) was applied to assist statistical methods in the analysis of associated variations to disease susceptibility. A big data set was simulated using the published genotype frequencies of 26 SNPs amongst eight genes for breast cancer. Results showed that the proposed DBM-PSO successfully determined two- to six-order models of gene-gene interaction for the risk association with breast cancer (odds ratio > 1.0; *P* value <0.05). Analysis results supported that the proposed DBM-PSO can identify good models and provide higher chi-square values than conventional PSO. This study indicates that DBM-PSO is a robust and precise algorithm for determination of gene-gene interaction models for breast cancer.

## 1. Introduction

Genome-wide association studies (GWAS) for the analysis of gene-gene interaction are important fields for detecting the effects of cancer and disease [1–4]. Such studies usually entail the collection of a vast number of samples and SNPs selected from several related genes of disease in order to identify the association amongst genes. Disease effect, in general, is influenced by the best association between SNPs from several genes; these SNPs could have a potential association to provide information for disease analysis. Therefore, a method for searching high-order interactions is needed to determine the potential association between several loci.

Good models of the association between SNPs from several genes are usually hidden in the large number of possible models. The sum of all possible models of association between case data and control data can be computed by *C*(*n*, *m*) × *g* ^*m*^, where *n* represents a total number of SNPs, *m* is a selected number of SNPs, and *g* is the number of genotypes. Data mining and machine learning methods have been proposed for use in GWAS data analysis. These computational approaches were developed to examine epistasis in family-based and case-control association studies [5–12]. The genetic algorithm (GA), particle swarm optimization (PSO), and chaotic particle swarm (CPSO) methods were proposed to identify the models of gene-gene interaction. However, the ability to determine the relative model quality needs to be improved. In mathematics, the problem space for identifying good models is not linear and the algorithm converges easily to a local optima, since no better models are found near the best model in that region. PSO often leads to premature convergence, especially in complex multipeak search problems. Therefore, the use of chaotic sequences to improve the PSO has been proposed to identify models of gene-gene interaction [7]. An improved PSO using a double-bottom chaotic maps (DBM-PSO) [13] has been shown to overcome the respective disadvantages of PSO and CPSO. In this study, DBM-PSO is applied to assist statistical methods in the analysis of associated variations to disease susceptibility.

A total of 26 SNPs obtained from eight related genes of breast cancer (EGF, IGF1, IGF1R, IGF2, IGFBP3, IL10, TGFB1, and VEGF) were used to test the various methods for comparison of the association models. It is proposed that the interactions between polymorphisms of breast cancer-related genes may have synergistic effects on the pathogenesis of cancer and disease; this would explain differences in disease susceptibility. The quality of a model of gene-gene interaction can be assessed by determining its odds ratio (OR), confidence intervals, and *P* value. We systematically evaluate the model effects from two- to five-order interactions to compare the DBM-PSO with other PSOs methods.

## 2. Methods

### 2.1. Problem Description

To identify the quality of the models of gene-gene interaction problem, the model includes SNPs and their corresponding genotypes. The set *X* = {*x*_1_, *x*_2_, *x*_3_, …, *x*_*D*_} represents a possible model as a solution in the problem space; each parameter *x* is a real number. The chi-square test is used to design the PSO and DBM-PSO fitness functions. The objective is to search for a vector X* which has its own best fitness value according to the evaluation of fitness function *f*(*X*)(*f* : *δ*⊆*R* ^*D*^ → *R*); that is, *f*(X*) > *f*(*X*), for all *X* ∈ *δ*, where *δ* is a nonempty large finite set serving as the search space and *δ* = *R* ^*D*^.

### 2.2. Particle Swarm Optimization

Particle swarm optimization (PSO) is a population-based stochastic optimization technique [14]. The conception of PSO is based on a robust theory of swarm intelligence to search for an optimal resolution of complex problems. Swarm intelligence describes an automatically evolving system based on simulating the social behaviour of organisms, for example, knowledge sharing. Therefore, valuable information can be shared amongst swarm members to suggest a common objective which leads individuals toward an optimal direction. PSO has been used to solve several types of optimization problems [15], including function optimization and parameter optimization [16] and shows promise for nonlinear function optimization [17–22]. In PSO, possible solutions are represented as the particles. During generation, particle positions are adjusted according to the updated velocity toward a significant objective. The objective of each particle is defined based on the particle's previous experience (*pbest*) and knowledge commonly held by the population (*gbest*). Thus, particles can effectively converge into a solution-rich area to find the better solution. Finally, the particles follow the current best particle in the search space until a predefined number of generations are reached. The PSO procedure entails (1) population initialization, (2) objective function evaluation, (3) identification of *pbest* and *gbest*, (4) particle updating, and (5) the termination condition. These steps are described in detail in the following section.

### 2.3. Double-Bottom Map Particle Swarm Optimization

Double-bottom map particle swarm optimization (DBM-PSO) was proposed by Yang et al. in 2012 [13]. While PSO is easily complicated by the existence of nonlinear fitness function with multiple local optima, this is not an issue for DBM-PSO. A local optima, *f*_*i*_ = *f*(*X*_*i*_), can be described as ∃*ε* > 0  ∀ *X* ∈ *δ* : ||*X* − *X*_*i*_|| < *ε*⇒*f*(*X*) ≤ *f*_*i*_ ≤ *f*(X*), where ||·|| represents any *p*-norm distance measure. In PSO, the flexibilities of given constraints and vector space in the problem influence the determination of the best solution. Generally speaking, *r*_1_ and *r*_2_ independently influence search exploitation and exploration, and the effect of *r*_1_ and *r*_2_ on the convergence behaviour is very important in PSO. Recently, chaos approaches have been proposed to overcome the inherent disadvantages of PSO. Chaotic maps are easily applied in PSO to prevent entrapment of the population in a local optima [23]. DBM-PSO proposes a new type of chaotic map, called double-bottom maps, to improve the search ability of PSO. Double-bottom maps are used to design an updating function to balance the exploration and exploitation for PSO search capability. The superiority of the double-bottom map over other chaotic maps lies in the fact that it provides high frequencies in the three regions over time, that is, 0.0, 0.5, and 1.0. Ideally, the distribution ratios of 0.0, 0.5, and 1.0 can be effective in balancing the search behaviour; however, the double-bottom map is designed to satisfy this PSO property.

Algorithm 1 shows the DBM-PSO pseudocode and explains all processes in DBM-PSO to identify the best model of gene-gene interaction. The difference between PSO and DBM-PSO is that the proposed double-bottom map is applied in the updating function of the PSO process (symbol 14 of Algorithm 1). All steps in DBM-PSO for identifying the models of gene-gene interaction problems are explained below.

### 2.4. Initializing Particles and DBMr

In DBM-PSO, a point in the search space is a set which includes the real element *x*,  *x* ∈ *R*. Each particle is a possible solution to the corresponding problem. The subsequent iteration is denoted by *i* = 0, 1, …, Iteration_max⁡_. Since the elements in a set are likely to change over a sequence of iterations, (1) represents the *j*th particle in the population of *i*th iteration as

(1) $$\begin{array}{c} X_{j,i}\;=\;\left\{x_{j,i,1},x_{j,i,2},\ldots ,x_{j,i,D}\;|\;x\;\in \;R\right\}. \end{array}$$

In this study, a particle in the population represents a solution, that is, a model of gene-gene interaction. A particle contains two separate sets: a set of selected SNPs and a set of genotypes. For each element in *X*_*j*_, a certain range within the value is restricted. The values are related to physical components or measurement, that is, natural bounds. The initial population (at *i* = 0) process covers a certain range as much as possible by uniformly randomizing individuals within the search space constrained according to the minimum and maximum bounds, which are represented by *SNP*_min⁡_ and *SNP*_max⁡_ and *Genotype*_min⁡_ and *Genotype*_max⁡_, respectively. Equation (2) shows all genotypes. The homozygous reference genotype is represented as 1, while the heterozygous genotype is represented as 2, and the homozygous variant genotype is represented as 3:

(2) $$\begin{array}{c} \text{Genotype}=\{\begin{array}{cc} 1, & \text{AA}\;\text{type},\\ 2, & \text{Aa}\;\text{type},\\ 3, & \text{aa}\;\text{type}. \end{array} \end{array}$$

The particles are generated by (3). Particles are initialized by generating the random set in a particle:

(3) $$\begin{array}{c} x_{j,d}=\{\begin{array}{cc} \text{Random}\left(SNP_{\min},SNP_{\max}\right), & d\leq \frac{D}{2}\\ \text{Random}\left(Genotype_{\min},Genotype_{\max}\right), & d>\frac{D}{2}, \end{array} \end{array}$$

where *SNP*_max⁡_ and *SNP*_min⁡_ represent a limited SNP, while *Genotype*_max⁡_ and *Genotype*_min⁡_ represent the limited possible genotypes. For example, let *X*_*j*,0_ = (1, 3, 4, 2, 1, 2); thus *X*_*j*,0_ represents the *j*th  *X* in the first generation (at *i* = 0) of selected SNPs (1, 3, 4) and genotypes (2, 1, 2) and can be described by the SNPs associated with the genotypes as follows: (1, 2), (3, 1), and (4, 2).

All random values (DBMr) in the particles are generated with a random value between 0.0 and 1.0 for each independent run.

### 2.5. Evaluating the Qualities of Particles Using Fitness Function

In the DBM-PSO process, the fitness function measures the quality of particles in the population. The studies of gene-gene interaction focus on the combinations of SNP genotypes to identify the highest chi-square (*χ* ^2^) value between breast cancer cases and noncancer cases; the value is called the fitness value in DBM-PSO.  Algorithm 2 shows the fitness value computation pseudocode. In (4) and (5), symbols *p* and *n* are, respectively, the sizes of case data and control data, while in (4), (5), (6), and (7), *P* and *N* are, respectively, the sets of case data and control data. The *a* in (4) is used to count the number of *P* including the *X*_*j*_; that is, *X*_*j*_⊆*P*_*k*_. The *b* in (5) is used to count the number of *N* including the *X*_*i*_; that is, *X*_*j*_⊆*N*_*k*_. The *c* in (6) represents the total number of unmatched *X*_*j*_ in the *P*; that is, *X*_*j*_⊄*P*_*k*_. The *d* in (7) represents the total number of unmatched *X*_*j*_ in the *N*; that is, *X*_*j*_⊄*N*_*k*_. Equation (9) computes the difference between case data and control data and is used to determine whether the model is associated with risk or protection. Equation (10) is used to compute the fitness value if the objective is to search the risk association model. Equation (11) is used to compute the fitness value if the objective is to search the protection association model. Equation (12) is the chi-square (*χ* ^2^) function and is used to compute the *χ* ^2^ value between breast cancer cases and noncancer cases in this study. Consider

(4) $$\begin{array}{c} a=f\left(X_{j}\right)=\overset{p}{\underset{k=1}{\sum }}u\left(X_{j},P_{k}\right), \end{array}$$

(5) $$\begin{array}{c} b=f\left(X_{j}^{}\right)=\overset{n}{\underset{k=1}{\sum }}u\left(X_{j},N_{k}\right), \end{array}$$

(6) $$\begin{array}{c} c=p-a, \end{array}$$

(7) $$\begin{array}{c} d=n-b, \end{array}$$

where

(8) $$\begin{array}{c} u\left(X_{j},A\right)=\{\begin{array}{cc} 1, & \forall x\subseteq A,\\ 0, & \forall x⊄A, \end{array}\;\forall x\in X_{j} \end{array}$$

(9) $$\begin{array}{c} RorP=\frac{100}{\left(p\times n\right)\left(n\times a-p\times b\right)}, \end{array}$$

(10) $$\begin{array}{c} \text{fitness}\_\text{risk}=\{\begin{array}{cc} 0, & RorP<1,\\ \chi^{2}, & RorP>1, \end{array} \end{array}$$

(11) $$\begin{array}{c} \text{fitness}\_\text{protection}=\{\begin{array}{cc} 0, & RorP>1,\\ \chi^{2}, & RorP<1, \end{array} \end{array}$$

(12) $$\begin{array}{c} \chi^{2}=\frac{\left(a+b+c+d\right){\left(a\times d-b\times c\right)}^{2}}{\left(a+b\right)\left(c+d\right)\left(a+c\right)\left(b+d\right)}. \end{array}$$

### 2.6. Updating the *pbest* *s* of Particles and *gbest* of Population

Each particle can be improved according to the two objectives, *pbest* and *gbest*, to search for a better solution. *pbest*_*j*_ indicates the best value of a position previously visited by the *j*th particle, and its position is denoted by *P*_*j*_ = (*p*_*j*,1_, *p*_*j*,2_, …, *p*_*j*,*d*_). Equations (13) are the updating functions for a particle's *pbest* position and *pbest* value, respectively, as follows:

(13) Pj={Xj,f(Xj)≥pbestj,Pj,f(Xj)<pbestj,pbestj={f(Xj),f(Xj)≥pbestj,pbestj,f(Xj)<pbestj,

where *gbest* indicates the best value of all *pbest* values for a particle and its position is denoted by *G* = (*g*_1_, *g*_2_, …, *g*_*d*_). Equations (14) provide the updating function for *gbest* position and *gbest* value, respectively, as follows:

(14) $$\begin{array}{c} G=\{\begin{array}{cc} P_{j}, & pbest_{j}\geq gbest,\\ G, & pbest_{j}<gbest, \end{array}\\ gbest=\{\begin{array}{cc} pbest_{j}, & pbest_{j}\geq gbest,\\ gbest, & pbest_{j}<gbest. \end{array} \end{array}$$

### 2.7. Updating Particle Velocities and Positions

DBM-PSO executes a search for optimal solutions by continuously updating particle positions in all iterations. Equations (15) and (16) are used to update the velocity and a position of the *j*th particle, respectively, as follows:

(15) vj,dnew=w×vj,dold+c1×DBMrj,1×(pj,d−xj,dold)+c2×DBMrj,2×(gd−xj,dold),

(16) xj,dnew=xj,dold+vj,dnew,

where *c*_1_ and *c*_2_ are acceleration constants that control how far a particle moves in a given iteration. Random values, DBM*r*_*j*,1_ and DBM*r*_*j*,2_, in (15) are generated by a function based on the results of the double-bottom map with values between 0.0 and 1.0; they are described in the following section. Velocities *v*_*j*,*d*_^new^ and *v*_*j*,*d*_^old^ are a particle's new and old velocities, respectively. Positions *x*_*j*,*d*_^old^ and *x*_*j*,*d*_^new^ are the particle's current and updated positions, respectively. Variable *w* is the inertia weight and is described in the following section.

### 2.8. Updating Particle Inertia Weight Values

Variable *w* in DBM-PSO is called the inertia weight which is used to control the impact of a particle's previous velocity. Throughout all iterations, *w* decreases linearly from 0.9 to 0.4 [24], and the equation can be written as

(17) $$\begin{array}{c} w=\left(w_{\max}-w_{\min}\right)\times \frac{\text{Iteratio}{\text{n}}_{\max}-\text{Iteratio}{\text{n}}_{i}}{\text{Iteratio}{\text{n}}_{\max}}+w_{\min}, \end{array}$$

where Iteration_*i*_ represents the *i*th iteration and Iteration_max⁡_ represents the iteration size. Values *w*_max⁡_ and *w*_min⁡_ represent the maximal and minimal values of *w*, respectively.

### 2.9. Updating Particle DBM*r* Values

In DBM-PSO, two random values in the updating function are generated by the following double-bottom map function:

(18) $$\begin{array}{c} {\text{DBM}r}_{j,t+1}\;=\;\frac{\left[\sin\left(4\pi {\text{DBM}r}_{j,t}\right)\;+\;1\right]}{2}.\;\; \end{array}$$

### 2.10. Parameter Settings

In this study, all methods used the same parameters to test the search ability for the identification of the models of gene-gene interaction. The population size is 100 and the maximal iteration is 100. The value of inertia weight *w* is set from 0.9 to 0.4 [25]. Both learning factors, *c*_1_ and *c*_2_, are equal to 2 [26]. All tests are implemented in Java as a single thread in a PC environment running 32-bit Windows 7 with an Intel coreTM2 Quad CPU Q6600 at 2.4 GHz and 4 GB of RAM.

### 2.11. Statistical Analysis

The model of associations between SNPs can be evaluated by odds ratio (OR) and its 95% CI and *P* value [27]. OR can evaluate the models to quantitatively measure the risk of disease; *P* value can evaluate whether the results are statistically significant for the difference between the case data and control data. All statistical analyses are implemented using SPSS version 19.0 (SPSS Inc., Chicago, IL).

## 3. Results and Discussion

### 3.1. Data Set

The growth factor-related genes of breast cancer, including genes of EGF, IGF1, IGF1R, IGF2, IGFBP3, IL10, TGFB1, and VEGF with 26 SNPs, were tested in this study. A genotype generator is used to generate a large simulated data set according to the genotype frequencies.  Algorithm 3 shows the genotype generator pseudocode to explain how the data set was generated. The genotype frequencies of SNPs are collected from Pharoah et al.'s breast cancer association study [39], which explains the significance of these SNPs of genes in breast cancer.

### 3.2. Evaluation of Breast Cancer Susceptibility Using 26 SNPs from Eight Growth Factor-Related Genes

Table 1 shows the performance (OR and 95% CI) for estimating the effect of a single SNP from eight growth factor-related genes (EGF, IGF1, IGF1R, IGF2, IGFBP3, IL10, TGFB1, and VEGF). Amongst the 26 SNPs in the eight genes, eight SNPs in four genes display a statistically significant OR  (*P* < 0.05) for breast cancer. Six SNPs have a risk (OR > 1.0) association for breast cancer, including rs5742678-GG, rs1549593-AA, rs6220-GG, IGFIR-10-aa, rs2132572-GA and -AA, and rs1800470-CC. The highest and lowest OR values are 1.33 and 1.09, respectively. Two SNPs have a protection (OR < 1.0) association for breast cancer, including rs2229765-AA and rs2854744-CC. The highest and lowest OR values are 0.88 and 0.82, respectively. The other SNPs show no statistically significant OR for breast cancer.

### 3.3. Analysis of Models for Gene-Gene Interaction with Risk Association between the Case and Control Data Sets Using PSO, CPSO, and DBM-PSO

Table 2 shows the 2- to 7-order risk association models for gene-gene interaction. The results are compared with the *χ* ^2^ value, with a high value indicating a good result. The model of 2-SNPs with their corresponding genotypes, SNPs (1, 7) with genotypes 1-3, [rs5742678-CC]-[IGF1R-10-aa], is identified as having 9.451 *χ* ^2^ value to explain the difference between the case and control data sets for three methods. However, the results of 3- to 7-SNPs clearly indicate that the DBM-PSO algorithm exhibited an improved search ability over PSO and CPSO in terms of the comparison with the *χ* ^2^ value. For example, in 3-SNPs, DBM-PSO is identified as having a *χ* ^2^ value of 8.772, but those of PSO and CPSO are 3.364 and 3.997, respectively. Table 2 shows the (OR) and its 95% CI, which estimate the impact of the risk association model on the occurrence of breast cancer. A bigger OR value (>1) indicates a stronger risk association between the SNPs with combined genotypes and the disease. DBM-PSO shows high OR (1.346–10.018) values for models with a high association for the risk of breast cancer, and the *P* value (<0.05) indicates that the models have a statistically significant difference between patients and nonpatients. Aside from a 3-SNP model of CPSO, the *P* values of models in 3- to 7-SNPs of PSO and CPSO show no statistical significance, indicating that PSO and CPSO have difficulty in identifying statistically significant models for risk association for breast cancer. However, DBM-PSO successfully identifies good models for risk association for breast cancer.

### 3.4. Analysis of Models of Gene-Gene Interaction with Protection Association between Case and Control Data Sets Using PSO, CPSO, and DBMPSO

Table 3 shows the 2- to 7-order protection association models. The OR values (<1) estimate the impact of the protection association model on the occurrence of breast cancer. High *χ* ^2^ values in the models indicate good results, and the *P* value (<0.05) indicates that the model has a statistically significant difference between patients and nonpatients. The results of 3- to 7-SNPs show that DBM-PSO possesses higher *χ* ^2^ values than PSO and CPSO, indicating that DBM-PSO is better to search for good protection association models than other methods. DBM-PSO has OR values ranging from 0.755 to 0.850, with a *P* value of <0.05 for protection with breast cancer. The 2-SNP and 3-SNP models in PSO and CPSO show a statistically significant difference between patients and nonpatients (*P* < 0.05), and the 4-SNP model in CPSO also shows a statistically significant difference. Although CPSO provides better OR values than DBM-PSO in the 5-, 6-, and 7-SNP models, the *P* values indicate that these models are not statistically significant. DBM-PSO successfully identifies good models for protection association for breast cancer.

### 3.5. Discussion

Effects between SNPs from several genes could contribute to disease development. Case-control studies are the main method to determine the association between SNPs. Many breast cancer studies have analysed the associations between important related genes [28–34], hypothesizing that disease risk may be associated with the cooccurrence of SNPs displaying a jointed effect, including genes related to DNA repair [35, 36], chemokine ligand-receptor interactions [37], and estrogen-response genes [4].

Evolutionary algorithms are applied to identify good models of gene-gene interaction [7, 9]. Previous studies have used the difference between case and control data sets to design the fitness function, allowing for the identification of models with high difference values for all SNP combinations. However, the highest difference between the case and control data sets is not necessarily statistically significant (*P* < 0.05). The chi-square test is a statistical tool to evaluate the difference between the observed and expected data sets under specific hypothetical conditions. A property of the chi-square test is that the chi-square value is inversely proportional to *P* value. Therefore, the chi-square test is used to design the fitness function in this study. PSO and CPSO [7] were used to search for good models based on the new fitness function, but the results (Tables 2 and 3) fail to identify high-order associations. However, DBM-PSO effectively identified good risk and protection association models of gene-gene interactions for breast cancer. Statistical methods, such as *P* value, OR, and its 95% CI, provide strong validation of the search ability of DBM-PSO.

PSO and DBM-PSO use the fitness functional computation to calculate complexity. DBM-PSO can be observed in (15) and (18). Equation (18) is only used to amend the original PSO updating equation (15). Therefore, DBM-PSO does not increase the complexity of the PSO search process. The computational complexity of DBM-PSO is big-O(*nm*), where *n* is the number of iterations and *m* is the number of particles.

The results of DBM-PSO are influenced by its parameters, including double-bottom chaotic maps (18), population size, iteration size, and *c*_1_ and *c*_2_ in the updating function (15). Yang et al. [13] tested the 22 most commonly used representative benchmark functions, selecting the optimal parameters (4*π*) in the proposed double-bottom chaotic maps. Therefore, the parameter is suggested as 4*π* in (18). The population and iteration sizes could be adjusted according to the size of the data set. Population size suggested a setting from 50 to 200 and the suggested number of iterations ranges from 100 to 1000. *c*_1_ and *c*_2_ are both suggested to be 2 [38].

## 4. Conclusion

We proposed a new fitness function to identify good models of gene-gene interaction for the investigation of polygenic diseases and cancers. The fitness function based on chi-square test addresses the disadvantage of previously proposed fitness functions, in that the highest difference between the case and control data sets is not necessarily statistically significant (*P* < 0.05). Our proposed DBM-PSO showed to be able to successfully determine the 26 SNP cross interactions for risk and protection models of gene-gene interactions in breast cancer. The results indicate that DBM-PSO can successfully use the chi-square test to identify good models by evaluating the difference between the observed and expected data sets under specific hypothetical conditions.

## Figures and Tables

**Algorithm 1:**
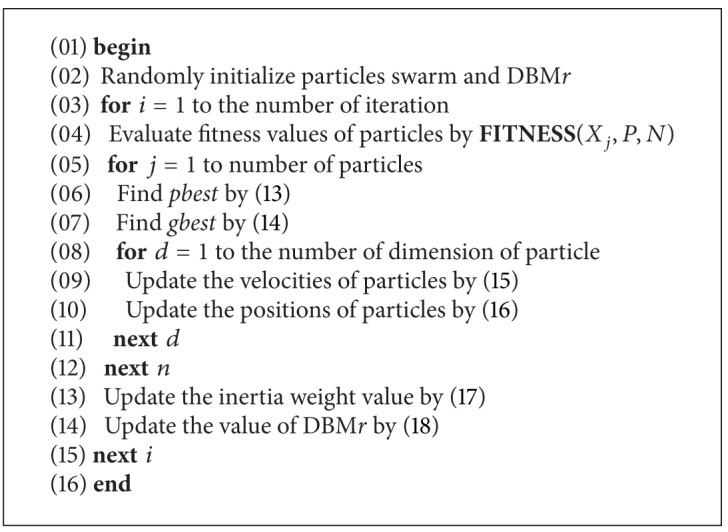
DBMPSO pseudocode.

**Algorithm 2:**
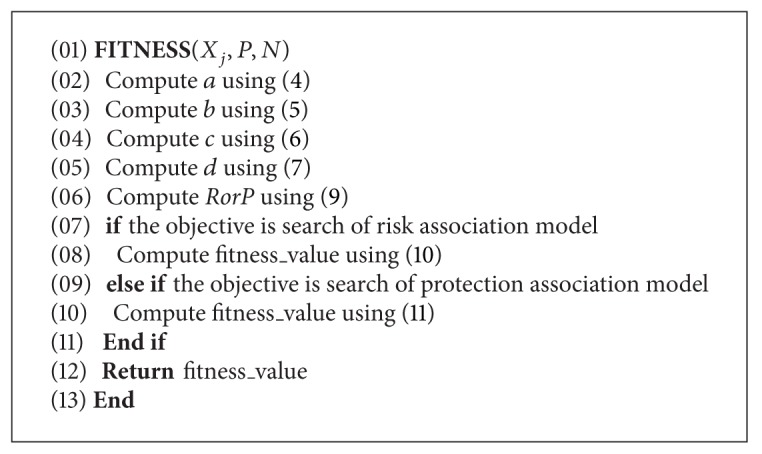
Fitness value computation pseudocode.

**Algorithm 3:**
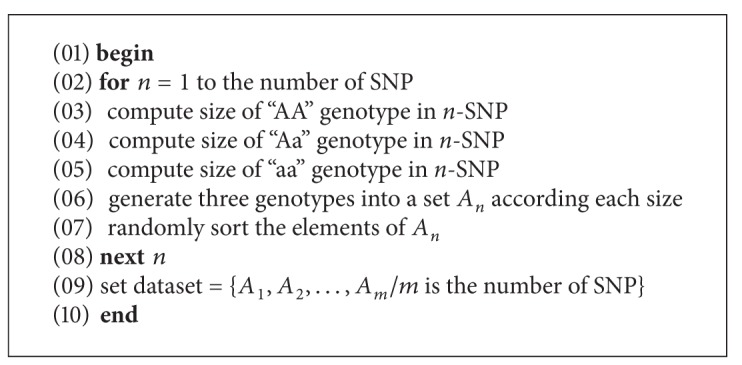
Genotype generator pseudocode.

**Table 1:** Estimated effect (odds ratio and 95% CI) from individual SNPs of 26 growth factor-related genes on the occurrence of breast cancer patients.

SNP (Genes)^a ^	SNP type	Case number/ normal number^a^	Odds ratio	95% CI
1. rs2237054 (EGF)	1-TT	4408/4418		
	2-TA	570/569	1.00	0.89–1.14
	3-AA	22/13	1.70	0.85–3.37

2. rs5742678 (IGF1)	1-CC	2797/2866		
	2-CG	1844/1837	1.03	0.95–1.12
	3-GG	359/297	1.24	1.05–1.46

3. rs1549593 (IGF1)	1-CC	2924/2970		
	2-CA	1753/1771	1.01	0.93–1.09
	3-AA	323/259	1.27	1.07–1.50

4. rs6220 (IGF1)	1-AA	2643/2698		
	2-AG	1933/1951	1.01	0.93–1.10
	3-GG	424/351	1.23	1.06–1.44

5. rs2946834 (IGF1)	1-CC	2295/2336		
	2-CT	2171/2150	1.03	0.95–1.12
	3-TT	534/514	1.06	0.93–1.21

6. rs1568502 (IGF1R)	1-AA	2914/2955		
	2-AG	1840/1807	1.03	0.95–1.12
	3-GG	246/238	1.05	0.87–1.26

7. IGF1R-10 (IGF1R)	1-AA	3169/3201		
	2-Aa	1545/1582	0.99	0.91–1.08
	3-aa	286/217	1.33	1.11–1.60

8. rs2229765 (IGF1R)	1-GG	1523/1429		
	2-GA	2533/2489	0.96	0.87–1.05
	3-AA	944/1082	0.82	0.73–0.92

9. rs8030950 (IGF1R)	1-CC	2737/2745		
	2-CA	1902/1917	1.00	0.92–1.08
	3-AA	361/338	1.07	0.92–1.25

10. rs680 (IGF2)	1-GG	2538/2451		
	2-GA	2074/2183	0.92	0.85–1.00
	3-AA	388/366	1.02	0.88–1.19

11. rs3741211 (IGF2)	1-TT	1936/1971		
	2-TC	2367/2269	1.06	0.98–1.16
	3-CC	697/760	0.93	0.83–1.05

12. IGF2-05 (IGF2)	1-AA	2651/2694		
	2-Aa	1955/1952	1.02	0.94–1.11
	3-aa	394/354	1.13	0.97–1.32

13. IGF2-06 (IGF2)	1-AA	2160/2162		
	2-Aa	2237/2284	0.98	0.90–1.07
	3-aa	603/554	1.09	0.96–1.24

14. rs2132571 (IGFBP3)	1-GG	2415/2407		
	2-GA	2163/2157	1.00	0.92–1.09
	3-AA	422/436	0.97	0.83–1.12

15. rs2471551 (IGFBP3)	1-GG	3225/3284		
	2-GC	1591/1515	1.07	0.98–1.17
	3-CC	184/201	0.93	0.76–1.15

16. rs2854744 (IGFBP3)	1-AA	1538/1469		
	2-AC	2487/2475	0.96	0.88–1.05
	3-CC	975/1056	0.88	0.79–0.99

17. rs2132572 (IGFBP3)	1-GG	2908/3027		
	2-GA	1805/1728	1.09	1.00–1.18
	3-AA	287/245	1.22	1.02–1.46

18. rs3024496 (IL10)	1-TT	1218/1235		
	2-TC	2533/2549	1.01	0.92–1.11
	3-CC	1249/1216	1.04	0.93–1.17

19. rs1800872 (IL10)	1-CC	3059/3017		
	2-CA	1660/1722	0.95	0.87–1.03
	3-AA	281/261	1.06	0.89–1.27

20. rs1800890 (IL10)	1-TT	1703/1701		
	2-TA	2455/2508	0.98	0.90–1.07
	3-AA	842/791	1.06	0.95–1.20

21. rs1554286 (IL10)	1-CC	3400/3446		
	2-CT	1431/1410	1.03	0.94–1.12
	3-TT	169/144	1.19	0.95–1.49

22. rs1800470 (TGFB1)	1-TT	1850/1914		
	2-TC	2372/2399	1.02	0.94–1.11
	3-CC	778/687	1.17	1.04–1.32

23. rs699947 (VEGF)	1-CC	1236/1273		
	2-CA	2511/2463	1.05	0.95–1.16
	3-AA	1253/1264	1.02	0.91–1.14

24. rs1570360 (VEGF)	1-GG	2278/2341		
	2-GA	2214/2132	1.07	0.98–1.16
	3-AA	508/527	0.99	0.87–1.13

25. rs2010963 (VEGF)	1-GG	2354/2279		
	2-GC	2133/2157	0.96	0.88–1.04
	3-CC	513/564	0.88	0.77–1.01

26. rs3025039 (VEGF)	1-CC	3744/3741		
	2-CT	1160/1174	0.99	0.90–1.08
	3-TT	96/85	1.13	0.84–1.52

^a^Data collected from the literature [39].

**Table 2:** Estimation of the best risk model of gene-gene interaction on the occurrence of breast cancer as determined by PSO, CPSO, and DBMPSO.

	Combined SNP	SNP genotypes	Cases number	Controls number	*χ* ^2^ value	Odds ratio	95% CI	*P* value
2-SNP								
PSO	1,7	1-3	259	195	9.451	1.346	1.11–1.63	0.002
		Other	4741	4805				
CPSO	1,7	1-3	259	195	9.451	1.346	1.11–1.63	0.002
		Other	4741	4805				
DBMPSO	1,7	1-3	259	195	9.451	1.346	1.11–1.63	0.002
		Other	4741	4805				
3-SNP								
PSO	2,14,25	3-1-1	84	62	3.364	1.361	0.98–1.89	0.068
		Other	4916	4938				
CPSO	1,6, 7	1-1-3	148	116	3.997	1.285	1.00–1.64	0.046
		Other	4850	4884				
DBMPSO	7,11,21	3-2-1	93	57	**8.772**	**1.644**	**1.18**–**2.29**	**0.003**
		Other	4907	4943				
4-SNP								
PSO	1,14,20,23	3-3-1-2	2	0	1.000	3.001	0.31–28.86	0.341
		Other	4998	5000				
CPSO	1,4, 11,14	1-3-2-1	86	67	2.396	1.289	0.93–1.78	0.123
		Other	4914	4933				
DBMPSO	1,7, 11,21	1-3-2-1	87	53	**8.374**	**1.653**	**1.17**–**2.33**	**0.004**
		Other	4913	4947				
5-SNP								
PSO	2,7, 15,18,24	1-3-1-3-2	15	8	2.135	1.878	0.80–4.43	0.151
		Other	4985	4992				
CPSO	3,10,17,24,26	3-1-1-3-1	9	3	3.004	3.004	0.81–11.10	0.099
		Other	4991	4997				
DBMPSO	1,2, 7,11,21	1-1-3-2-1	49	27	**6.417**	**1.823**	**1.14**–**2.92**	**0.013**
		Other	4951	4973				
6-SNP								
PSO	2,6, 8,16,18,25	3-1-1-2-3-2	3	1	1.000	3.001	0.31–28.86	0.341
		Other	4997	4999				
CPSO	2,11,16,18,22,23	1-2-1-2-3-2	14	9	1.089	1.557	0.67–3.60	0.301
		Other	4986	4991				
DBMPSO	1,2, 7,10,11,21	1-1-3-1-2-1	27	12	**6.417**	**2.257**	**1.14**–**4.46**	**0.019**
		Other	4973	4988				
7-SNP								
PSO	1,3, 6,12,21,24,26	1-3-2-1-3-1-1	2	0	1.000	3.001	0.31–28.86	0.341
		Other	4998	5000				
CPSO	1,2, 3,9, 19,21,24	1-1-3-1-1-2-3	4	1	1.801	4.002	0.45–35.82	0.215
		Other	4996	4999				
DBMPSO	1,3, 5,9, 17,23,24	1-3-2-1-2-2-1	10	1	**7.372**	**10.018**	**1.28**–**78.29**	**0.028**
		Other	4990	4999				

**Table 3:** Estimation of the best protection model of gene-gene interaction on the occurrence of breast cancer as determined by PSO, CPSO, and DBMPSO.

	Combined SNP	SNP genotypes	Cases number	Controls number	*χ* ^2^ value	Odds ratio	95% CI	*P* value
2-SNP								
PSO	1,8	1-3	816	941	10.789	0.841	0.76–0.93	0.001
		Other	4184	4059				
CPSO	1,8	1-3	816	941	10.789	0.841	0.76–0.93	0.001
		Other	4184	4059				
DBMPSO	1,8	1-3	816	941	10.789	0.841	0.76–0.93	0.001
		Other	4184	4059				
3-SNP								
PSO	8,9, 22	3-1-2	225	269	4.123	0.829	0.69–0.99	0.043
		Other	4775	4731				
CPSO	3,8, 9	1-3-1	319	371	4.209	0.850	0.73–0.99	0.040
		Other	4681	4629				
DBMPSO	1,8, 15	1-3-1	527	624	**9.238**	**0.826**	**0.73**–**0.94**	**0.002**
		Other	4473	4376				
4-SNP								
PSO	4,8, 14,22	2-3-1-2	76	99	3.077	0.764	0.57–1.03	0.080
		Other	4924	4901				
CPSO	10,17,21,23	2-1-1-1	223	268	4.337	0.824	0.69–0.99	0.038
		Other	4777	4732				
DBMPSO	1,10,17,21	1-2-1-1	692	795	**8.381**	**0.850**	**0.76**–**0.95**	**0.004**
		Other	4308	4205				
5-SNP								
PSO	5,6, 8,9, 26	1-1-3-2-1	75	91	1.568	0.821	0.60–1.12	0.211
		Other	4925	4909				
CPSO	2,4, 8,11,18	1-2-3-1-2	32	44	1.909	0.726	0.46–1.15	0.169
		Other	4968	4956				
DBMPSO	1,2, 6,8, 15	1-1-1-3-1	167	218	**7.026**	**0.758**	**0.62**–**0.93**	**0.008**
		Other	4833	4782				
6-SNP								
PSO	4,8, 15,19,22,24	1-3-2-2-1-3	0	2	1.000	0.333	0.04–3.20	0.341
		Other	5000	4998				
CPSO	3,4, 12,16,20,24	1-1-1-2-2-3	21	28	1.005	0.749	0.43–1.32	0.318
		Other	4979	4972				
DBMPSO	1,10,15,17,21,26	1-2-1-1-1-1	327	394	**6.710**	**0.818**	**0.70**–**0.95**	**0.010**
		Other	4673	4606				
7-SNP								
PSO	5,8, 11,13,14,24,25	1-1-3-1-1-2-1	3	6	1.001	0.500	0.13–2.00	0.327
		Other	4997	4994				
CPSO	10,12,16,17,19,22,26	2-2-2-1-2-2-1	20	27	1.047	0.740	0.41–1.32	0.308
		Other	4980	4973				
DBMPSO	1,10,13,15,17,21,26	1-2-2-1-1-1-1	141	185	**6.139**	**0.755**	**0.60**–**0.94**	**0.014**
		Other	4859	4815				
